# Dexamethasone and supportive care with or without whole brain radiotherapy in treating patients with non-small cell lung cancer with brain metastases unsuitable for resection or stereotactic radiotherapy (QUARTZ): results from a phase 3, non-inferiority, randomised trial

**DOI:** 10.1016/S0140-6736(16)30825-X

**Published:** 2016-10-22

**Authors:** Paula Mulvenna, Matthew Nankivell, Rachael Barton, Corinne Faivre-Finn, Paula Wilson, Elaine McColl, Barbara Moore, Iona Brisbane, David Ardron, Tanya Holt, Sally Morgan, Caroline Lee, Kathryn Waite, Neil Bayman, Cheryl Pugh, Benjamin Sydes, Richard Stephens, Mahesh K Parmar, Ruth E Langley

**Affiliations:** a Northern Centre for Cancer Care, Newcastle Hospitals NHS Foundation Trust, Newcastle upon Tyne, UK; b Medical Research Council Clinical Trials Unit at University College London, London, UK; c Queen's Centre for Oncology and Haematology, Castle Hill Hospital, Hull, UK; d Institute of Cancer Sciences, The University of Manchester, Manchester Academic Health Science Centre, The Christie NHS Foundation Trust, Manchester, UK; e Bristol Haematology and Oncology Centre, Bristol, UK; f Newcastle Clinical Trials Unit and Institute of Health and Society, Newcastle University, Newcastle upon Tyne, UK; g Wales Cancer Research Network, Cardiff UK; h The Beatson West of Scotland Cancer Centre, Greater Glasgow Health Board and Clyde, Glasgow, UK; i Patient representative, Manchester, UK; j Trans Tasman Radiation Oncology Group, Waratah, NSW, Australia; k University of Queensland, QLD, Australia; l Nottingham University Hospital, Nottingham, UK; m Weston Park Hospital, Sheffield, UK; n Queen Elizabeth Hospital, Kings Lynn, UK

## Abstract

**Background:**

Whole brain radiotherapy (WBRT) and dexamethasone are widely used to treat brain metastases from non-small cell lung cancer (NSCLC), although there have been no randomised clinical trials showing that WBRT improves either quality of life or overall survival. Even after treatment with WBRT, the prognosis of this patient group is poor. We aimed to establish whether WBRT could be omitted without a significant effect on survival or quality of life.

**Methods:**

The Quality of Life after Treatment for Brain Metastases (QUARTZ) study is a non-inferiority, phase 3 randomised trial done at 69 UK and three Australian centres. NSCLC patients with brain metastases unsuitable for surgical resection or stereotactic radiotherapy were randomly assigned (1:1) to optimal supportive care (OSC) including dexamethasone plus WBRT (20 Gy in five daily fractions) or OSC alone (including dexamethasone). The dose of dexamethasone was determined by the patients' symptoms and titrated downwards if symptoms improved. Allocation to treatment group was done by a phone call from the hospital to the Medical Research Council Clinical Trials Unit at University College London using a minimisation programme with a random element and stratification by centre, Karnofsky Performance Status (KPS), gender, status of brain metastases, and the status of primary lung cancer. The primary outcome measure was quality-adjusted life-years (QALYs). QALYs were generated from overall survival and patients' weekly completion of the EQ-5D questionnaire. Treatment with OSC alone was considered non-inferior if it was no more than 7 QALY days worse than treatment with WBRT plus OSC, which required 534 patients (80% power, 5% [one-sided] significance level). Analysis was done by intention to treat for all randomly assigned patients. The trial is registered with ISRCTN, number ISRCTN3826061.

**Findings:**

Between March 2, 2007, and Aug 29, 2014, 538 patients were recruited from 69 UK and three Australian centres, and were randomly assigned to receive either OSC plus WBRT (269) or OSC alone (269). Baseline characteristics were balanced between groups, and the median age of participants was 66 years (range 38–85). Significantly more episodes of drowsiness, hair loss, nausea, and dry or itchy scalp were reported while patients were receiving WBRT, although there was no evidence of a difference in the rate of serious adverse events between the two groups. There was no evidence of a difference in overall survival (hazard ratio 1·06, 95% CI 0·90–1·26), overall quality of life, or dexamethasone use between the two groups. The difference between the mean QALYs was 4·7 days (46·4 QALY days for the OSC plus WBRT group *vs* 41·7 QALY days for the OSC group), with two-sided 90% CI of −12·7 to 3·3.

**Interpretation:**

Although the primary outcome measure result includes the prespecified non-inferiority margin, the combination of the small difference in QALYs and the absence of a difference in survival and quality of life between the two groups suggests that WBRT provides little additional clinically significant benefit for this patient group.

**Funding:**

Cancer Research UK, Medical Research Council Clinical Trials Unit at University College London, and the National Health and Medical Research Council in Australia.

## Introduction

In 2012, 1·82 million cases of lung cancer were diagnosed worldwide.^1^ Overall, up to 30% of patients with non-small cell lung cancer (NSCLC) will present with or develop brain metastases subsequently;^2^, ^3^ for patients with stage 3 disease treated with radical intent who achieve a partial or complete radiological response, the risk of subsequently developing brain metastases is 50%.^4^, ^5^ Lung cancer is the most common cause of brain metastases, constituting 50–65% of patients within published epidemiological studies and brain radiotherapy trials.^6^, ^7^, ^8^, ^9^, ^10^, ^11^ Historically, survival rates after the development of metastatic brain disease in patients with NSCLC have been consistently lower than for patients with other primary cancer sites such as breast cancer^9^ and range from 2 to 6 months,^12^, ^13^

**Research in context**

**Evidence before this study**

Whole brain radiotherapy (WBRT) is widely used for the treatment of brain metastases from non-small cell lung cancer (NSCLC). We searched PubMed and the abstracts of major conferences (such as the American Society of Clinical Oncology) using the search terms “brain metastases”, “irradiation (or radiotherapy)”, and “steroids (or corticosteroids)”, with no constraints imposed on the timeframe for the search, for randomised evidence to support this practice. We found only one relevant randomised clinical trial, which recruited 48 patients with brain metastases from various primary cancers, and concluded that WBRT offered only limited benefit and its use as standard practice was difficult to justify. We identified no trials done solely in patients with NSCLC.

**Added value of this study**

This is the only adequately powered randomised clinical trial assessing the use of WBRT in patients with brain metastases from NSCLC. Although overall the patients recruited in this study had a poorer prognosis than those in previous case series, which had provided the evidence base for the use of WBRT in this setting, the trial population reflects the typical clinical experience, in which very few patients meet the criteria for the best prognostic classes.

**Implications of all the available evidence**

The combined evidence suggests that WBRT offers no substantial benefit to most patients with brain metastases from NSCLC in terms of improved survival, overall quality of life, or reduction in steroid use. The implication for clinical care is that optimal supportive care (OSC) is as effective as OSC plus WBRT, and the implication for future research is that potential new treatments could be assessed in addition to OSC, rather than in addition to, or in place of, WBRT.

The practice of giving whole brain radiotherapy (WBRT) in combination with steroids (usually dexamethasone) is a widely used approach in the management of patients with brain metastases based mainly on reports from the 1950s–60s.^14^, ^15^ However, a Cochrane review^16^ in 2012 only identified one randomised controlled trial (RCT), done by the Eastern Cooperative Oncology Group (ECOG) and published in 1971, specifically addressing the efficacy of supportive care plus WBRT compared with supportive care alone. This study randomised only 48 patients with multiple primary tumour sites (30 with lung cancer; the number with NSCLC as opposed to small-cell lung cancer was not specified) to prednisone only or prednisone plus WBRT.^17^ The primary outcome was clinical remission, assessed by the patients' neurological and performance status, with overall survival as a secondary outcome. Although the investigators concluded that the combination of prednisone and radiation offered a slight advantage compared with prednisone alone in terms of duration of remission and survival, they felt that overall this did not justify the use of WBRT as standard treatment because the survival benefit it offered was so small.

Nevertheless, WBRT remained standard practice, and in the 1970s and 1980s, dose-finding studies were done both in North America^6^, ^7^ and the UK^9^ to investigate the optimum WBRT regimen from the point of view of overall survival. These trials included patients with malignancies from all solid primary sites and thus by definition were extremely heterogeneous. In 2007, Rades and colleagues^18^ showed that 20 Gy in five fractions provided similar survival times for patients with NSCLC compared to more protracted regimens. In the UK, Europe, Australasia, and Canada, 20 Gy in five fractions is frequently adopted, whereas ten or more fractions are more commonly used in the USA.^19^, ^20^

In recent years, the potential treatment options for metastatic brain disease from NSCLC have continued to evolve and increasingly include neurosurgery, stereotactic radiosurgery, and systemic treatments. Thus far, only patients with a solitary brain metastasis have been shown to derive statistically significant survival benefit when WBRT is combined with focal (surgical or stereotactic) management of the intracranial disease.^21^ The absence of level 1 evidence for a survival improvement from focal management for patients with more than one brain metastasis might be obscured by the competing risk of death from extracranial disease.^22^

Survival outcomes for NSCLC patients with multiple brain metastases are poor with radiation, radiosurgery, or chemotherapy, alone or in combination, and have hardly changed since the original publications of the 1980s.^23^, ^24^ Thus, the historically developed treatment of steroids and WBRT continues to be a very widely used option, particularly when other methods are not feasible.^24^, ^25^, ^26^, ^27^, ^28^, ^29^, ^30^

There have been repeated calls for a definitive and sufficiently powered randomised controlled trial of supportive care plus WBRT versus supportive care alone.^31^, ^32^ In view of this and aware that the patients in our clinics with brain metastases from NSCLC appeared to fare much less well than hoped for when treated with WBRT, we designed the Quality of Life After Treatment for Brain Metastases (QUARTZ) trial to assess the efficacy of WBRT for the treatment of brain metastases from NSCLC. We postulated that omission of WBRT would not cause an important detriment in quality-adjusted life-years (QALYs). The non-inferiority design acknowledged a potential reduction in overall survival, but it was thought that this would be balanced by an absence of deterioration in quality of life as a result of radiation-induced fatigue, hair loss, nausea, and scalp discomfort, which would justify the omission of standard WBRT.

## Methods

### Study design and participants

QUARTZ is a multi-centre, randomised, non-inferiority, two-arm parallel-group phase 3 trial for patients with histologically proven NSCLC and brain metastases (confirmed by CT or MRI). This trial was done at 69 UK and three Australian hospitals. National ethical approval was obtained in the UK (through the North West Medical Research and Ethics Committee) and in Australia (through the Metro South Health Service District Human Research Ethics Committee), with local approval at each participating centre. The trial was designed to be both pragmatic and inclusive (patients with a Karnofsky Performance Status [KPS] of <70 were eligible). Clinicians were encouraged to approach potential participants about the trial if there was uncertainty in the clinicians' or patients' minds about the potential benefit of WBRT, and a multidisciplinary team that included both neurosurgeons and radiation oncologists had concluded that the patient was unsuitable for either surgery or stereotactic radiotherapy. Previous treatment with systemic anticancer treatment (chemotherapy or tyrosine kinase inhibitors [TKI]) was permitted (with predefined washout periods of 4 weeks for chemotherapy and 1 week for TKIs). Exclusion criteria included previous radiotherapy to the brain, or previous or current illness thought likely to interfere with protocol treatment. Subsequent or simultaneous (extracranial) palliative radiotherapy and systemic treatments post-randomisation were permitted at the clinician's discretion, because these were not thought to interfere with the efficacy of WBRT, and to withhold any further appropriate treatment would be unethical. Participants were aged 18 years or older, gave informed consent, and had to be able to respond to questions about their quality of life, symptoms, and side-effects in weekly telephone assessments. The trial was registered with ISRCTN, number ISRCTN3826061. The protocol is available online.

### Randomisation and masking

Participants were randomly assigned (1:1) to receive either optimal supportive care (OSC) plus WBRT or OSC alone. Allocation to treatment group was done by a phone call (made almost exclusively by the research nurse, who then usually followed up the patient) from the hospital to the Medical Research Council Clinical Trials Unit at University College London using a minimisation programme with a random element and stratification by centre, KPS, gender, status of brain metastases (newly diagnosed or progressive disease), and status of primary lung cancer (absent, controlled, or uncontrolled).

Patients and investigators were not blinded to their treatment allocation because to do so would have required giving them sham WBRT, which we did not believe was justifiable on clinical or ethical grounds. In addition, travel to receive sham treatments could have influenced the patient's quality of life, producing a biased assessment of quality of life and QALYs in those patients.

### Procedures

Patients were randomly assigned to receive either OSC plus WBRT or OSC alone. OSC included oral dexamethasone given with a proton pump inhibitor with the dose of steroid determined by the patients' symptoms and titrated downwards if symptoms improved, as well as support from a named specialist nurse and immediate access to specialised clinicians and palliative care teams. WBRT was defined as 20 Gy in five daily fractions ideally given over 5–8 days with a 4–8 MV linear accelerator with two parallel opposed fields, commenced as soon as was practical after randomisation.

Anticancer treatments, patient-reported symptoms and quality of life (using the EuroQol EQ-5D 3L questionnaire)^33^ were recorded before randomisation and through weekly telephone assessments or clinic visits for at least 12 weeks from randomisation and monthly thereafter. Data on carers' experiences and their perception of the patient's symptoms and quality of life were collected and will be reported separately.

### Statistical analysis

QUARTZ is a non-inferiority trial assessing the omission of WBRT with a primary outcome measure of QALYs. QALYs were chosen as the primary outcome measure because both quality of life and survival were considered to be key outcome measures for patients, their families, and treating physicians, and would provide the most useful interpretation of the data. Given the poor prognosis of this population, a relatively small boundary for assessing non-inferiority was needed. At the time of the design, following discussions within the Trial Management Group and with clinicians in the UK, we decided that a reduction in QALYs of no more than 1 week would convince the clinical and patient communities that WBRT could be omitted from standard practice. An initial estimate of 6 weeks for the median QALY in the WBRT plus OSC group based on a study by Gerrard and colleagues^34^ was used to obtain the original sample size of 1036 patients with a hazard ratio (HR) of 1·2, 80% power, and a one-sided 2·5% significance level.

Once 50 patients had been randomly assigned to the WBRT plus OSC group, a pre-planned review of the sample size was done. The average QALY in the WBRT plus OSC group was 5 weeks and the sample size was re-calculated. Simultaneously, owing to slow recruitment, the Trial Steering Committee approved changing the significance level to a one-sided 5% level, while retaining the 1-week non-inferiority boundary (and consequent HR of 1·25), resulting in a revised sample size of 534 patients. These changes were made blind to the accumulating results from the trial (ie without assessing data from the OSC only group).

We recognised that recruitment to QUARTZ would be challenging, primarily because of the absence of randomised data to inform and support discussion of the trial with patients and colleagues alike. 3 years after starting QUARTZ, and despite the reduction in target sample size, accrual rates remained below target and the Trial Management Group took the unusual step of formally asking the independent Trial Steering Committee if interim data from the initial cohort recruited to QUARTZ (151 patients) could be released.^35^ This decision was made blind to the accumulating results from the trial. After the interim release, the accrual rate improved and was maintained, reaching the required total in August, 2014.

Secondary outcome measures included overall survival and quality of life. QALYs were estimated and compared using the methods described by Billingham and Abrams.^36^ From each completed EQ-5D questionnaire, a utility score was generated, which ranged from 1 (where a patient reported no problems for all five questions) to −0·59 (where a patient reported severe problems for all five questions). The average utility score of all surviving uncensored patients was calculated at every point where an assessment took place, and multiplied by the value of the survivor function whenever either the survivor function or the average utility score changed. The estimated mean QALY was then calculated as the area under the resultant step curve for each treatment group, with CIs estimated using bootstrapping. Overall survival was calculated as the time from randomisation until death from any cause, with survivors having their data censored at the time they were last known to be alive. Treatment groups were compared with a stratified log-rank test and hazard ratios calculated with Cox proportional hazard models, with adjustment for the stratification factors used at randomisation. Median survival estimates were obtained from a flexible parametric survival model with adjustment for the same factors. Although the sample size calculation for this non-inferiority trial was based on a one-sided 5% significance level, the primary outcome results are presented with a two-sided 90% CI. The critical value for the one-sided 5% significance level and the upper bound of the two-sided 90% CI are equivalent. Quality of life, as reported using the EQ-5D 3L questionnaire, and KPS were each compared at 4, 8, and 12 weeks post-randomisation, using analysis of variance with adjustment for baseline values.

### Role of the funding source

The study was funded by Cancer Research UK and the Medical Research Council (UK), and the National Health and Medical Research Council (Australia). Cancer Research UK reviewed and approved the study design, and neither funder had any role in data collection, data analysis, data interpretation, or writing of the report. The corresponding author had full access to all the data in the study, and had final responsibility for the decision to submit for publication.

## Results

**Figure 1:**
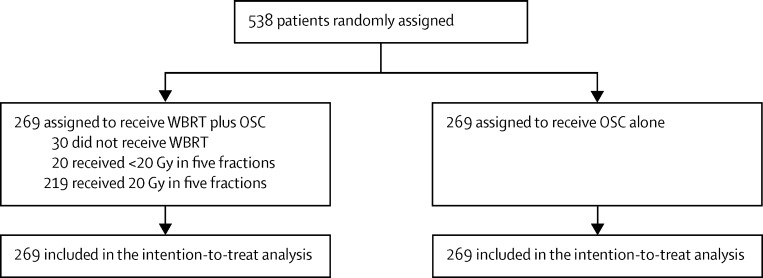
Trial profile WBRT=whole brain radiotherapy. OSC=optimal supportive care.

Between March 2, 2007, and Aug 29, 2014, 538 patients from 69 UK and three Australian centres were randomly assigned (1:1) to receive either OSC plus WBRT (269), or OSC alone (269). All randomly assigned patients were included in these analyses, on an intention-to-treat basis. Although an analysis using a per-protocol population is also customary in non-inferiority trials, we believe it is not appropriate here because only one group receives a formal treatment regimen and therefore omission of patients from any analysis based on them receiving treatments would probably bias results because poorly performing patients would only be removed from the WBRT group. More than 90% of the expected follow-up forms were received, with 80% of expected quality-of-life forms completely filled in, and one patient lost to follow-up (figure 1). The baseline characteristics of the two groups were well balanced (table 1), and data are available for all 538 patients unless otherwise stated: 314 (58%) were male, median age was 66 years (range 38–85 years), and 203 (38%) had a KPS of less than 70. Brain metastases were diagnosed within the 8 weeks before randomisation in 467 (87%) patients, and a solitary brain metastasis was present in 162 (30%) of 533 patients. Symptoms and quality of life at baseline were similar between the two groups (table 2).

**Figure 2:**
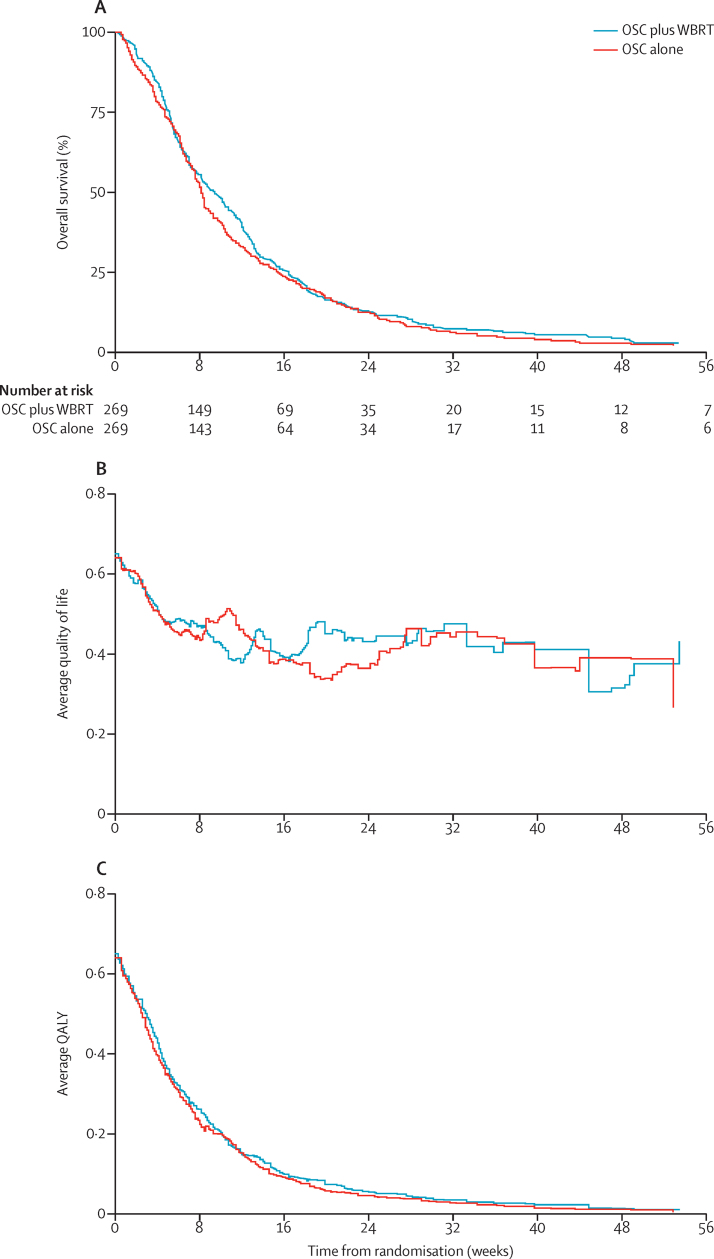
Components of quality-adjusted life-years (QALY) (A) Overall survival from randomisation. (B) Quality of life. The average utility score is calculated from all surviving and uncensored patients for every time at which any patient completed the EQ-5D questionnaire. If a patient has not completed the questionnaire on a particular day, their score is imputed by assuming a straight line connecting their closest utility scores before and after the day in question. (C) Quality-adjusted life-years. The survivor function is multiplied by the average utility score at each time that either the survivor function or the average utility score changes. The area under the resultant step function is the mean QALY for each treatment group. Graphs are only displayed up to 56 weeks for presentation purposes and due to the small number of patients beyond this point.

The primary outcome measure is QALYs, which combines overall survival and quality of life. The mean (SD) QALY for patients assigned to the OSC plus WBRT group was 46·4 days (3·66), and for those assigned to the OSC group was 41·7 days (3·23; figure 2C), a difference of −4·7 QALY days in favour of WBRT (90% CI of −12·7 to 3·3; appendix p 12).

At 4 weeks there was little difference in symptoms and side-effects between the two groups (appendix p 1). Data were collected for 20 different symptoms and side-effects. Those receiving OSC plus WBRT reported more moderate or severe episodes of drowsiness than those receiving OSC alone (67 [42%] of 159 *vs* 39 [28%] of 139, respectively, p=0·0151), hair loss (51 [34%] of 149 *vs* 1 [1%] of 137, respectively, p=0·0001), nausea (15 [10%] of 151 *vs* 3 [2%] of 139, respectively, p=0·0067), and dry or itchy scalp (11 (7%] of 148 *vs* 1 [1%] of 138, respectively, p=0·0057; appendix p 1).

There was little difference in the number of serious adverse events reported in the two groups (table 3). Overall, 89 patients receiving OSC plus WBRT and 82 patients receiving OSC reported at least one serious adverse event over the course of the trial. The most commonly reported events were infections, neurological problems, and pulmonary problems, with no evidence of any difference between groups in the rate of any event.

Quality-of-life assessments (with the EQ-5D) were generally well completed, with a median of six assessments per patient for those in the OSC plus WBRT group (IQR 3–11, range 1–36), and five assessments for those in the OSC alone group (IQR 3–10, range 1–28). Quality of life, as measured by the utility score generated from the EQ-5D 3L responses, remained similar over time (figure 2B), with no significant differences between the groups at 4, 8, or 12 weeks. The number of patients with maintained or improved quality of life compared with baseline was also similar between the groups at 4 weeks (81 [54%] of 149 patients receiving OSC plus WBRT *vs* 80 (57%) of 140 patients receiving OSC), 8 weeks (40 [44%] of 90 patients receiving OSC plus WBRT *vs* 40 [51%] of 78 patients receiving OSC), and 12 weeks (24 [44%] of 54 patients receiving OSC plus WBRT *vs* 21 [49%] of 43 patients receiving OSC).

KPS changes were similar between the two groups, with no significant differences at 4, 8, or 12 weeks (p=0·9272, p=0·2823, and p=0·0724, respectively), with average performance status decreasing slightly over time. The mean (SD) decline in KPS compared with baseline at 4, 8, and 12 weeks was 8·3 (13·89), 11·3 (13·03), and 18·0 (15·53), respectively, for patients receiving OSC plus WBRT, versus 8·5 (14·52), 13·4 (15·91), and 13·4 (13·66), respectively, for patients receiving OSC.

At randomisation 525 (98%) of 538 patients received dexamethasone (median 8 mg a day). By 4 weeks post-randomisation, 11 (5%) of 233 patients receiving OSC and 16 (7%) of 245 patients receiving OSC plus WBRT had spent some time off dexamethasone (p=0·4328), and this number increased to 24 (10%) of 233 and 30 (12%) of 245, respectively, at 8 weeks (p=0·5641). Most patients had their dexamethasone dose decreased compared with the initial dose given at randomisation: during the first 4 weeks 142 (61%) of 233 patients receiving OSC and 143 (58%) of 245 patients receiving WBRT plus OSC had their dose reduced (p=0·5771); during the first 8 weeks 153 (66%) of 233 patients receiving OSC and 167 (68%) of 245 patients receiving WBRT plus OSC had their dose reduced (p-=0·6268). Dexamethasone use over the first 8 weeks post-randomisation is shown in more detail in the appendix (p 5, 10, 11).

In the OSC plus WBRT group (n=269), 93 (35%) patients started radiotherapy within 7 days of randomisation, 97 (36%) within 8–14 days, and 49 (18%) after more than 14 days. 30 patients (11%) received no radiotherapy, 222 (83%) received 20 Gy, and 17 (6%) received less than 20 Gy. Of the 30 patients assigned to, but who did not receive WBRT, ten died before starting radiotherapy, 14 were considered too ill or their disease had progressed, five refused treatment (one of these patients did later receive WBRT 6 months after randomisation), and one could not be contacted. Of the 17 patients who received less than 20 Gy, two were treated using a 12 Gy in two fractions regimen in error, and the remaining 15 were considered too unwell to complete their treatment.

More patients reported receiving additional anticancer therapy in the OSC group (86 [35%] of 246 patients) than in the OSC plus WBRT group (54 [21%] of 259 patients, p=0·0005). This difference was predominantly due to an increase in the amount of radiotherapy given (55 [22%] of 246 patients receiving OSC compared with 27 [10%] of 259 patients receiving OSC plus WBRT), with the thorax being the most common site of treatment, although seven patients, assigned to receive OSC alone, also received WBRT. Use of chemotherapy, surgery, and TKIs was similar between the two groups: 42 patients received chemotherapy (17 receiving OSC plus WBRT and 25 receiving OSC), 29 received TKI (14 receiving OSC plus WBRT and 15 receiving OSC) and seven patients received both (three receiving OSC plus WBRT and four receiving OSC).

Overall survival in the two groups was similar (HR 1·06, 95% CI 0·90–1·26, p=0·8084; figure 2A). By the time of analysis, 536 of the 538 patients had died (267 in the OSC plus WBRT group and 269 in the OSC alone group) with a median survival (estimated from a flexible parametric model) of 9·2 weeks (95% CI 7·2–11·1) for patients who received OSC plus WBRT and 8·5 weeks (95% CI 7·1–9·9) for patients who received OSC. Of the two surviving patients, one was still participating in the trial, and one has been lost to follow-up. Cause of death was available for 531 patients, with 519 (98%) reported as having a disease-related death.

**Figure 3:**
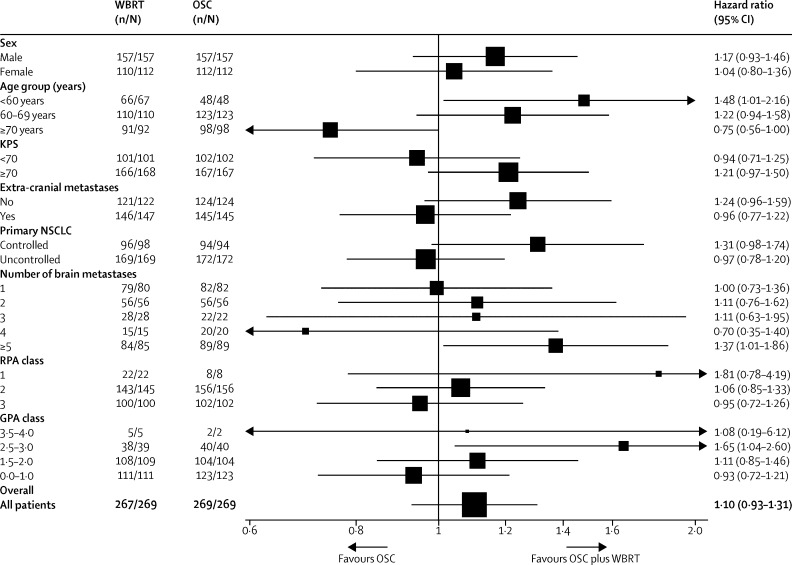
Forest plot of overall survival by patient characteristics All hazard ratios are obtained from Cox proportional hazard models with adjustment for randomised group only. KPS=Karnofsky Performance Status. NSCLC=non-small cell lung cancer. RPA=recursive partitioning analysis. GPA=graded prognostic assessment. WBRT=whole brain radiotherapy. OSC=optimal supportive care.

Analyses of the effect of WBRT on overall survival in different subgroups are presented in figure 3 and the appendix (p 6). A significant interaction was found between treatment group and age (p=0·0062), and a non-significant association between treatment group and KPS (p=0·0964) and status of NSCLC (p=0·0941), suggesting a possible difference in treatment effect across values of these factors. Younger patients, particularly those aged younger than 60 years, show improved survival with WBRT (figure 3, appendix p 6). The non-significant associations with KPS and primary NSCLC status suggested a potential survival benefit with WBRT for patients with KPS of at least 70, and those with controlled NSCLC. When assessing the predictive effect of the recursive partitioning analysis (RPA) and graded prognostic assessment (GPA) prognostic classes, there was no evidence that WBRT offered a survival advantage within any particular class (p=0·2762 for RPA and p=0·2642 for GPA). There were, however, non-significant associations which suggested a potential survival benefit with WBRT in the better prognostic groups (those with better reported survival) (p=0·0843 for RPA and p=0·0812 for GPA).

## Discussion

The QUARTZ trial is the sole adequately powered RCT specifically addressing the efficacy of supportive care plus WBRT compared with supportive care alone in patients with NSCLC. The data from the whole trial population suggest that WBRT can be omitted and patients treated with OSC alone, without an important reduction in either overall survival or quality of life. The estimate of the difference in QALYs was −4·7 days for the omission of WBRT. Similarly, there was just a 0·7 week (approximately 5 days) difference in median survival between the two groups, highlighting both the poor prognosis of this patient group and the limited benefit offered by WBRT.

The interaction analyses assessing the consistency of effect across subgroups (acknowledging that some of these were small and thus underpowered to detect differences, and only produce interactions of borderline statistical significance) suggest that the size of this effect might not be uniform across the full range of patient characteristics. Improved survival with WBRT was shown for younger patients, particularly those aged younger than 60 years (figure 3, appendix p 6). Other, non-significant, associations also suggested a potential survival benefit with WBRT for patients with good performance status and a controlled primary NSCLC, although WBRT did not show a statistically significant benefit in these latter two groups. Outside these groups (ie, older patients, those with poor performance status, or those with an uncontrolled primary NSCLC), WBRT appears to offer no benefit in terms of either survival or quality of life. These subgroups fall broadly in line with both the RPA^8^ and disease-specific Graded Prognostic Assessments (ds-GPA),^10^ both developed by the Radiation Therapy Oncology Group (RTOG). Since the RPA prognostic classes were described, the oncology community has broadly concluded that those patients who fell into the better RPA classes 1 and 2 required WBRT, whereas those within the poorest prognostic group (class 3, median survival 2·3 months) did not require WBRT, despite the absence of a solid evidence base to support this approach.^16^ Patients who fell within RPA class 3 were thus excluded from any subsequent brain metastases trials that involved radiotherapy.

In routine clinical practice, only 3·5–7·5% of patients with brain metastases fall into RPA class 1 and conversely, 40–50% fall into RPA class 3.^37^, ^38^, ^39^ Although the RPA class 3 patients have been excluded from clinical studies, clinicians continue to consider and frequently offer WBRT to this group because of the absence of alternative treatment options.^20^ The QUARTZ trial was the first opportunity to assess all these prognostic classes in a randomised setting, both in terms of their prognostic effect and their ability to predict WBRT benefit.

In line with everyday clinical experience, our data included only 30 (6%) of 533 RPA class 1 patients, and so we are unable to make any definitive statements about the benefit of WBRT in this group. We saw a non-significant association between RPA and treatment group, which suggested a potential benefit with WBRT with better RPA class, but further evidence is needed to firmly establish the size of any effect for patients who fell within RPA class 1. Importantly, patients who fell within RPA class 2 (301 [56%] of the 533 patients in the QUARTZ cohort), who have previously been thought to require and potentially benefit from WBRT, seemed to derive no clinically significant benefit from this treatment. In addition, QUARTZ has now provided data to back up the belief that WBRT should not be seen as a beneficial palliative treatment for patients falling within RPA class 3. Similarly, our data suggest that WBRT might still have a role for patients with the best prognoses according to GPA categories (those with scores of at least 2·5), but offers no benefit over supportive care alone for patients with lower scores and poorer prognoses.

A long-held belief is that treatment with WBRT allows patients to reduce and stop treatment with steroids. In our trial, the addition of WBRT to OSC was not associated with a significant reduction in dexamethasone dose or use in the first 8 weeks from randomisation, and challenges the dogma that WBRT can be seen as a potential steroid-sparing modality. At the time of randomisation, 373 (84%) of 443 patients had shown a clinical response to dexamethasone, and although the figure seen in the wider patient population might be expected to be slightly lower than this, it does serve as a reminder that steroids are highly effective at alleviating many of the symptoms experienced by patients at the time of presentation.

Importantly we have shown that randomised clinical trials within this population are possible, and have collected a large set of high quality data. QUARTZ was a very pragmatic trial, with few inclusion and exclusion criteria beyond there being clinician or patient uncertainty as to the benefit of WBRT. The trial did not mandate extra clinic visits, with most data being collected via telephone calls between the patient and their nurse, which resulted in a good amount of data completion despite this being required on a weekly basis. Although it is undeniably challenging to discuss clinical trials within a poor prognosis patient group, particularly if one option is to omit a common treatment and recruitment was slower than originally hoped, it is a credit to the patients, carers, nurses, and clinicians that these discussions did take place and recruitment to the trial was ultimately successful.

The primary outcome for QUARTZ was patient-reported QALYs. Given the potentially poor prognosis for this group of patients, this was considered the most appropriate measure. Hence, the EQ-5D tool was used. Although this is not a brain-specific quality-of-life tool, it does allow accurate calculation of a Health Utility Index which, when combined with overall survival, provides a measurement of QALY. Data from the EQ-5D responses showed that there was no evidence of a difference in quality of life, and detailed symptom data show only small differences in symptom patterns after treatment with WBRT (with increased levels of drowsiness, hair loss, nausea, and dry or itchy scalp). We decided not to overburden patients by collecting further quality-of-life data, but we acknowledge this as a limitation of the trial because we cannot provide a more disease-specific assessment of quality of life, as would have been provided by using a questionnaire such as the EORTC QLQ-BN20.^40^ Two further quality-of-life-related aspects of this trial have not been presented here, but will be discussed in detail in a separate publication. These involve data looking at the burden placed upon carers of patients within the trial, and the possibility of using a carer's assessment of the patient's quality of life as a proxy measure rather than asking the patient directly.

Another important point to be considered when discussing the trial results is that 11% of patients randomly assigned to receive WBRT did not receive any treatment, in the majority of cases because of rapid disease progression or death. We have not been able to find any factors from these patients that would have allowed them to be identified as likely to have disease progression or die before trial entry, and believe this reflects the clinical reality of this patient group, in which substantial deterioration can be rapid and unpredicted. Although this might be viewed as a criticism of the patient selection in the trial, it is potentially of value to both clinicians and patients, particularly when there is uncertainty about the value of WBRT.

The overall survival of patients in this trial was short, although not inconsistent with our predicted survival times on which we based the sample size calculations,^34^ and represents everyday clinical experience. Comparison with retrospective case series such as those published by the RTOG,^10^ for example where the median survival was around 7 months, is not straightforward for a number of reasons. First, such studies only include patients who receive WBRT, and so the 11% of patients in the WBRT group for whom WBRT was planned but could not be given, generally due to rapid decline, would have been excluded. Second, survival in such studies is usually measured from the date of diagnosis whereas in QUARTZ, the survival time is from the date of randomisation, with a median time between diagnosis and randomisation of 25 days. Finally, the figure of 7 months median survival is for all patients with NSCLC, including those who had surgery and stereotactic radiosurgery (ie, the patients with a better prognosis who were excluded from QUARTZ). The median survival in the RTOG series for patients who received WBRT alone was 3·42 months, which is much more similar to the figure seen in this trial.

During the first decade of the 21st century, local control using stereotactic radiotherapy or surgical resection of individual brain metastases emerged as a clinically beneficial treatment option for highly selected patients.^30^ For patients with potentially durable prognoses, WBRT is increasingly seen as a treatment provided in addition to this local control, or is held in reserve for salvage management should new or recurrent brain metastases develop at a later date, though without evidence from RCTs to support this evolving practice.^26^, ^30^, ^41^ Use of stereotactic radiotherapy continues to develop and is starting to be used in a wider patient population, for example in patients with a large number of brain metastases.^42^ Although still confined to the better prognosis patients (RPA 1 only), this development could further restrict the use of WBRT as a sole treatment.

The systemic treatment paradigms for patients with brain metastases from NSCLC have also developed because knowledge of the effect of mutational status has grown. Within the UK, routine epidermal growth factor receptor (EGFR) testing was introduced for patients with stage 3B and 4 NSCLC in 2010, and anaplastic lymphoma kinase (ALK) rearrangement testing became routine in 2012. Thus, during the recruitment period for QUARTZ, clinicians were increasingly able to specifically assess EGFR mutation and ALK-fusion status. Patients with such mutations (around 10% of patients for EGFR and 5% for ALK-rearrangement in the UK) plus metastatic brain disease might now be offered the appropriate systemic targeted treatment, frequently as the sole treatment, though their use does not preclude the use of WBRT.

In summary, QUARTZ provides compelling information for clinicians and patients alike; for younger patients, WBRT might offer a survival advantage, but for all other groups, omitting WBRT does not significantly affect QALY or overall survival.

## Figures and Tables

**Table 1:** Baseline characteristics

	**OSC plus WBRT (n=269)**	**OSC alone (n=269)**
**Sex**		
Men	157 (58%)	157 (58%)
Women	112 (42%)	112 (42%)
**Age (years)**		
Median	66	67
IQR	60–72	62–72
Range	38–84	45–85
Karnofsky Performance Status*		
30	2 (1%)	0 (0%)
40	5 (2%)	4 (1%)
50	26 (10%)	31 (12%)
60	68 (25%)	67 (25%)
70	59 (22%)	60 (22%)
80	65 (24%)	66 (25%)
90	39 (14%)	35 (13%)
100	5 (2%)	6 (2%)
<70	101 (38%)	102 (38%)
≥70	168 (62%)	167 (62%)
**Extracranial metastases**		
No	122 (45%)	124 (46%)
Yes†	147 (55%)	145 (54%)
**NSCLC histology**		
Adenocarcinoma	148 (55%)	138 (51%)
Squamous	53 (20%)	66 (25%)
Large cell	7 (3%)	5 (2%)
NSCLC NOS	61 (23%)	60 (22%)
Primary NSCLC status*		
Absent	11 (4%)	9 (3%)
Controlled	87 (33%)	85 (32%)
Uncontrolled	169 (63%)	172 (65%)
Brain metastases status*		
Newly diagnosed	222 (83%)	220 (82%)
Progressive disease	47 (17%)	49 (18%)
**Time since brain metastases diagnosis**		
≤2 weeks	73 (27%)	63 (23%)
≤ 4 weeks	92 (34%)	90 (33%)
≤6 weeks	51 (19%)	54 (20%)
>6 weeks	53 (20%)	62 (23%)
Median (days)	23	26
Range (days)	2–235	0–196
**RPA prognostic class**		
1	22 (8%)	8 (3%)
2	145 (54%)	156 (59%)
3	100 (37%)	102 (38%)
Data unavailable	2	3
**GPA prognostic class**		
3·5–4·0	5 (2%)	2 (1%)
2·5–3·0	39 (15%)	40 (15%)
1·5–2·0	109 (41%)	104 (39%)
0·0–1·0	111 (42%)	123 (46%)
Data unavailable	5	0

Data are n (%) unless otherwise specified. All percentages calculated out of number of patients with non-missing data, rather than the number of patients randomly assigned. NSCLC=non-small cell lung cancer. PS=performance status. NOS=not otherwise specified. WBRT=whole brain radiotherapy. OSC=optimal supportive care. RPA=recursive partitioning analysis. GPA=graded prognostic assessment.

* Indicates variables used to stratify randomisation.

† Includes 42 patients receiving OSC plus WBRT and 41 patients receiving OSC noted to have liver metastases.

**Table 2:** Baseline symptoms and quality of life (moderate or severe)

	**OSC plus WBRT (n=269)**	**OSC alone (n=269)**
**Symptoms**		
Tiredness	107 (40%)	117 (44%)
Insomnia	74 (28%)	94 (35%)
Weakness	67 (25%)	80 (30%)
Drowsiness	63 (24%)	73 (27%)
Mood changes	55 (21%)	46 (17%)
Impaired sight	51 (19%)	45 (17%)
Memory loss	36 (13%)	36 (13%)
Dizziness	29 (11%)	38 (14%)
Weight loss	30 (11%)	32 (12%)
Confusion	21 (8%)	31 (12%)
Headache	27 (10%)	25 (9%)
Steroid-related cosmetic side-effects	25 (9%)	25 (9%)
Indigestion	21 (8%)	28 (10%)
Impaired speech	21 (8%)	26 (10%)
Weight gain	20 (8%)	24 (9%)
Thrush	19 (7%)	11 (4%)
Nausea	11 (4%)	9 (3%)
Seizures	8 (3%)	7 (3%)
Hair loss	8 (3%)	6 (2%)
Dry or itchy scalp	2 (1%)	4 (2%)
Any moderate or severe symptom	194 (72%)	209 (78%)
**Quality of life questions**		
Mobility	190 (71%)	187 (70%)
Self-care	93 (35%)	100 (37%)
Usual activities	192 (72%)	178 (67%)
Pain or discomfort	138 (52%)	113 (42%)
Anxiety or depression	119 (45%)	121 (45%)
Any problems in quality of life	237 (88%)	248 (93%)

Symptoms are ordered according to descending prevalence at randomisation. Percentages are calculated for patients with non-missing data, not all randomised patients. Symptoms were reported on a four-point scale: none, mild, moderate, or severe. Quality of life was reported on a three-point scale: no problems, some problems, severe problems. Patients are classified as having moderate or severe quality of life issues if they provide any of the following answers: mobility (“some problems walking about” or “confined to bed”); self-care (“some problems with washing or dressing” or “unable to wash or dress”); usual activities (“some problems performing usual activities” or “unable to perform usual activities”); pain or discomfort (“moderate pain or discomfort” or “extreme pain or discomfort”); and anxiety or depression (“moderately anxious or depressed” or “extremely anxious or depressed”). WBRT=whole brain radiotherapy. OSC=optimal supportive care.

**Table 3:** Serious adverse events

	**WBRT plus OSC (n=269)**	**OSC alone (n=269)**	p value*
Any serious adverse event	89 (33%)	82 (30%)	0·5786
Blood or bone marrow	0	1 (<1%)	1·000
Cardiac	2 (1%)	1 (<1%)	1·000
Constitutional	5 (2%)	3 (1%)	0·7245
Endocrine	1 (<1%)	0 (0%)	1·000
Gastric	1 (<1%)	7 (3%)	0·0683
Haemorrhage	2 (1%)	1 (<1%)	1·000
Infection	17 (6%)	16 (6%)	1·000
Lymphatic	0	1 (<1%)	1·000
Metabolic or laboratory	0	3 (1%)	0·2486
Musculoskeletal or soft tissue	1 (<1%)	4 (1%)	0·3727
Neurology	18 (7%)	31 (12%)	0·0713
Ocular or visual	0	1 (<1%)	1·000
Pain	4 (1%)	3 (1%)	1·000
Pulmonary or upper respiratory	22 (8%)	12 (4%)	0·1096
Renal or genitourinary	1 (<1%)	0	1·000
Vascular	6 (2%)	4 (1%)	0·7516
Other	1 (<1%)	0	1·000

All serious adverse events received were reviewed clinically, and body systems assigned according to Common Terminology Criteria for Adverse Events version 3.0. WBRT=whole brain radiotherapy. OSC=optimal supportive care.

* p values are calculated using Fisher's exact test.
