# Supplementary material for: Dexamethasone and supportive care with or without whole brain radiotherapy in treating patients with non-small cell lung cancer with brain metastases unsuitable for resection or stereotactic radiotherapy (QUARTZ): results from a phase 3, non-inferiority, randomised trial
Source: Lancet. 2016 Oct 22;388(10055):2004–14. doi: 10.1016/S0140-6736(16)30825-X (PMC5082599; doi:10.1016/S0140-6736(16)30825-X)
Supplement: Supplementary appendix [file mmc1.pdf]

THE LANCET

## Supplementary appendix

This appendix formed part of the original submission and has been peer reviewed. We post it as supplied by the authors.

Supplement to: Mulvenna P, Nankivell M, Barton R, et al. Dexamethasone and supportive care with or without whole brain radiotherapy in treating patients with non-small cell lung cancer with brain metastases unsuitable for resection or stereotactic radiotherapy (QUARTZ): results from a phase 3, non-inferiority, randomised trial. *Lancet* 2016; published online Sept 4. http://dx.doi.org/10.1016/S0140-6736(16)30825-X.

**Supplementary Tables and Figures**

**Supplementary Table S1: Change in symptoms from randomisation to four weeks.**

| **Symptom** | Severity at Baseline | OSC + WBRT (N=152) | | OSC Alone (N=139) | | P-value |
|---|---|---|---|---|---|---|
| | | Severity at 4 weeks | | Severity at 4 weeks | | |
| | | None/mild | Mod/Sev | None/mild | Mod/Sev | |
| Sight | None/mild | 120 (79%) | 10 (7%) | 103 (74%) | 15 (11%) | 0.2227 |
| | Moderate/severe | 16 (11%) | 5 (3%) | 14 (10%) | 7 (5%) | |
| | | | | | | |
| Speech | None/mild | 130 (86%) | 14 (9%) | 120 (86%) | 10 (7%) | 0.5578 |
| | Moderate/severe | 4 (3%) | 3 (2%) | 7 (5%) | 2 (1%) | |
| | | | | | | |
| Tired | None/mild | 59 (39%) | 40 (27%) | 52 (37%) | 35 (25%) | 0.6381 |
| | Moderate/severe | 15 (10%) | 36 (24%) | 21 (15%) | 31 (22%) | |
| | | | | | | |
| Drowsiness | None/mild | 83 (55%) | 49 (27%) | 83 (60%) | 30 (22%) | 0.0151 |
| | Moderate/severe | 9 (6%) | 18 (12%) | 17 (12%) | 9 (6%) | |
| | | | | | | |
| Insomnia | None/mild | 88 (58%) | 23 (15%) | 80 (58%) | 16 (12%) | 0.5879 |
| | Moderate/severe | 24 (16%) | 16 (11%) | 27 (19%) | 16 (12%) | |
| | | | | | | |
| Confusion | None/mild | 126 (83%) | 13 (9%) | 122 (88%) | 9 (6%) | 0.6815 |
| | Moderate/severe | 10 (7%) | 2 (1%) | 6 (4%) | 2 (1%) | |
| | | | | | | |
| Memory | None/mild | 114 (76%) | 16 (11%) | 115 (83%) | 8 (6%) | 0.0860 |
| | Moderate/severe | 15 (10%) | 5 (3%) | 13(9%) | 2 (1%) | |

| **Symptom** | Severity at Baseline | OSC + WBRT<br><br>(N=152) | | OSC Alone<br><br>(N=139) | | P-value |
|---|---|---|---|---|---|---|
| | | Severity at 4 weeks | | Severity at 4 weeks | | |
| | | | | | | |
| Mood | None/mild | 109 (73%) | 15 (10%) | 102 (74%) | 18 (13%) | 0.3385 |
| | Moderate/severe | 19 (13%) | 6 (4%) | 10 (7%) | 8 (6%) | |
| | | | | | | |
| Dizziness | None/mild | 124 (83%) | 12 (8%) | 113 (81%) | 7 (5%) | 0.7157 |
| | Moderate/severe | 7 (5%) | 7 (5%) | 11 (8%) | 8 (6%) | |
| | | | | | | |
| Seizures | None/mild | 147 (98%) | 1 (1%) | 133 (96%) | 4 (3%) | 0.1989 |
| | Moderate/severe | 2 (1%) | 0 (0%) | 2 (1%) | 0 (0%) | |
| | | | | | | |
| Weakness | None/mild | 86 (57%) | 33 (22%) | 84 (60%) | 21 (15%) | 0.3777 |
| | Moderate/severe | 14 (9%) | 18 (12%) | 15 (11%) | 19 (14%) | |
| | | | | | | |
| Headache | None/mild | 129 (85%) | 7 (5%) | 119 (86%) | 9 (6%) | 0.8350 |
| | Moderate/severe | 10 (7%) | 5 (3%) | 8 (6%) | 3 (2%) | |
| | | | | | | |
| Nausea | None/mild | 133 (88%) | 14 (9%) | 134 (96%) | 3 (2%) | 0.0067 |
| | Moderate/severe | 3 (2%) | 1 (1%) | 2 (1%) | 0 (0%) | |
| | | | | | | |
| Indigestion | None/mild | 134 (90%) | 7 (5%) | 119 (86%) | 7 (5%) | 0.4783 |
| | Moderate/severe | 7 (5%) | 1 (1%) | 9 (6%) | 4 (3%) | |
| | | | | | | |
| Hair loss | None/mild | 95 (64%) | 48 (32%) | 133 (97%) | 1 (1%) | 0.0001 |

| **Symptom** | Severity at Baseline | OSC + WBRT (N=152) | | OSC Alone (N=139) | | P-value |
|---|---|---|---|---|---|---|
| | | Severity at 4 weeks | | Severity at 4 weeks | | |
| | Moderate/severe | 3 (2%) | 3 (2%) | 3 (2%) | 0 (0%) | |
| | | | | | | |
| Weight gain | None/mild | 127 (86%) | 8 (5%) | 118 (86%) | 7 (5%) | 0.8346 |
| | Moderate/severe | 9 (6%) | 4 (3%) | 7 (5%) | 6 (4%) | |
| | | | | | | |
| Weight loss | None/mild | 132 (89%) | 6 (4%) | 118 (86%) | 7 (5%) | 1.000 |
| | Moderate/severe | 8 (5%) | 3 (2%) | 12 (9%) | 1 (1%) | |
| | | | | | | |
| Appearance | None/mild | 128 (86%) | 9 (6%) | 121 (87%) | 7 (5%) | 0.5437 |
| | Moderate/severe | 5 (3%) | 6 (4%) | 7 (5%) | 4 (3%) | |
| | | | | | | |
| Dry/Itchy scalp | None/mild | 137 (93%) | 10 (7%) | 135 (98%) | 1 (1%) | 0.0057 |
| | Moderate/severe | 0 (0%) | 1 (1%) | 2 (1%) | 0 (0%) | |
| | | | | | | |
| Thrush | None/mild | 132 (89%) | 8 (5%) | 125 (90%) | 10 (7%) | 1.000 |
| | Moderate/severe | 4 (3%) | 4 (3%) | 2 (1%) | 2 (1%) | |

Notes:

For each symptom, the question posed was “Has the patient experienced any problems in the following areas?”, with possible responses “None”, “Mild”, “Moderate”, or “Severe”.

Patients with data at both baseline and four weeks are summarised. Percentages are calculated from patients with non-missing data.

P-values are from Fisher’s Exact Test, and compare the number of patients reporting moderate or severe symptoms at four weeks.

Example: For hair loss in the OSC+WBRT arm, in total 143/149 (96%) patients had no/mild hair loss problems at baseline, and 98/149 (66%) had no/mild hair loss problems at 4 weeks. In more detail: 95 (64%) patients had no/mild problems at both baseline and 4 weeks; 48 (32%) had no/mild problems at baseline but a moderate/sever problem at 4 weeks; 3 (2%) had a moderate/severe problem at baseline but no/mild problems at 4 weeks; and 3 (2%) had a moderate/severe problem at both baseline and 4 weeks.

**Supplementary Table S2: Dexamethasone dose changes**

| During first… | Dexamethasone… | | WBRT + OSC<br><br>N=245 | OSC alone<br><br>N=233 | P-value |
|---|---|---|---|---|---|
| 4 weeks | Stopped | Yes | 16 (7%) | 11 (5%) | 0·4358 |
| | | No | 229 (93%) | 222 (95%) | |
| | | | | | |
| | Reduced | Yes | 143 (58%) | 142 (61%) | 0·5771 |
| | | No | 102 (42%) | 91 (39%) | |
| | | | | | |
| 8 weeks | Stopped | Yes | 30 (12%) | 24 (10%) | 0·5641 |
| | | No | 215 (88%) | 209 (90%) | |
| | | | | | |
| | Reduced | Yes | 167 (68%) | 153 (66%) | 0·6268 |
| | | No | 78 (32%) | 80 (34%) | |

Note: Dexamethasone is said to have been stopped if the patient spent any time not taking it during the four or eight weeks after randomisation, even if they subsequently started taking it again. Similarly, dexamethasone is said to have been reduced if the patient spent any time receiving a lower dose than that reported at randomisation, even if the dose was subsequently raised again.

Note: Treatment arms are compared using Fisher's Exact Test.

**Supplementary Table S3: Subgroup survival times.**

| Subgroup | Value | Statistic | WBRT + OSC | OSC alone |
|---|---|---|---|---|
| Sex | Male | N | 157 | 157 |
| | | Median OS | 9.4 | 7.9 |
| | | 95% CI | 7.0, 11.3 | 6.6, 8.9 |
| | | | | |
| | Female | N | 112 | 112 |
| | | Median OS | 9.4 | 8.4 |
| | | 95% CI | 7.1, 11.9 | 7.6, 10.3 |
| | | | | |
| Age Group | <60 | N | 67 | 48 |
| | | Median OS | 10.4 | 7.6 |
| | | 95% CI | 6.3, 13.4 | 4.6, 10.1 |
| | | | | |
| | 60 – 69 | N | 110 | 123 |
| | | Median OS | 11.0 | 7.9 |
| | | 95% CI | 8.7, 12.6 | 6.4, 8.4 |
| | | | | |
| | 70+ | N | 92 | 98 |
| | | Median OS | 7.0 | 9.3 |
| | | 95% CI | 5.9, 9.4 | 7.7, 11.4 |
| | | | | |
| KPS | <70 | N | 101 | 102 |
| | | Median OS | 5.7 | 5.7 |
| | | 95% CI | 5.0, 7.0 | 4.4, 7.3 |
| | | | | |

| Subgroup | Value | Statistic | WBRT + OSC | OSC alone |
|---|---|---|---|---|
| | 70+ | N | 168 | 167 |
| | | Median OS | 12.1 | 9.7 |
| | | 95% CI | 10.4, 13.4 | 8.3, 11.4 |
| | | | | |
| Extra-cranial metastases | No | N | 122 | 124 |
| | | Median OS | 10.4 | 8.4 |
| | | 95% CI | 7.9, 12.1 | 7.7, 9.7 |
| | | | | |
| | Yes | N | 147 | 145 |
| | | Median OS | 8.4 | 7.6 |
| | | 95% CI | 6.7, 10.3 | 6.4, 8.9 |
| | | | | |
| Primary NSCLC | Controlled | N | 98 | 94 |
| | | Median OS | 12.1 | 8.3 |
| | | 95% CI | 10.4, 15.1 | 7.6, 11.4 |
| | | | | |
| | Uncontrolled | N | 169 | 172 |
| | | Median OS | 7.7 | 7.7 |
| | | 95% CI | 6.4, 9.3 | 6.7, 8.4 |
| | | | | |
| Number of brain metastases | 1 | N | 80 | 82 |
| | | Median OS | 8.9 | 8.3 |
| | | 95% CI | 7.0, 11.9 | 6.9, 9.9 |
| | | | | |

| Subgroup | Value | Statistic | WBRT + OSC | OSC alone |
|---|---|---|---|---|
| | 2 | N | 56 | 56 |
| | | Median OS | 8.3 | 7.4 |
| | | 95% CI | 5.6, 10.7 | 6.3, 9.0 |
| | | | | |
| | 3 | N | 28 | 22 |
| | | Median OS | 9.6 | 10.1 |
| | | 95% CI | 5.3, 13.1 | 4.7, 13.7 |
| | | | | |
| | 4 | N | 15 | 20 |
| | | Median OS | 6.4 | 11.7 |
| | | 95% CI | 4.1, 13.7 | 4.7, 19.0 |
| | | | | |
| | 5+ | N | 85 | 89 |
| | | Median OS | 11.1 | 8.1 |
| | | 95% CI | 8.1, 13.0 | 6.4, 9.7 |
| | | | | |
| RPA | I | N | 22 | 8 |
| | | Median OS | 19.9 | 8.1 |
| | | 95% CI | 13.1, 48.7 | 1.6, 34.3 |
| | | | | |
| | II | N | 145 | 156 |
| | | Median OS | 11.3 | 9.0 |
| | | 95% CI | 9.6, 13.0 | 8.3, 11.0 |
| | | | | |
| | III | N | 100 | 102 |

| Subgroup | Value | Statistic | WBRT + OSC | OSC alone |
|---|---|---|---|---|
| | | Median OS | 5.7 | 5.7 |
| | | 95% CI | 5.0, 7.1 | 4.4, 7.3 |
| | | | | |
| GPA | 3.5 – 4.0 | N | 5 | 2 |
| | | Median OS | 11.9 | 7.6 |
| | | 95% CI | 1.0, Undef | 7.6, Undef |
| | | | | |
| | 2.5 – 3.0 | N | 39 | 40 |
| | | Median OS | 18.4 | 8.9 |
| | | 95% CI | 10.1, 23.4 | 8.0, 12.9 |
| | | | | |
| | 1.5 – 2.0 | N | 109 | 104 |
| | | Median OS | 8.1 | 8.0 |
| | | 95% CI | 6.7, 10.7 | 6.6, 9.3 |
| | | | | |
| | 0.0 – 1.0 | N | 111 | 123 |
| | | Median OS | 8.7 | 8.0 |
| | | 95% CI | 5.9, 11.1 | 6.4, 10.1 |

Note: KPS = Karnofsky Performance Status; RPA = Recursive Partitioning Analysis; GPA = Graded Prognostic Assessment.

Note: P-values for interactions between factors and treatment arm are: Sex 0.4477; Age group 0.0061(0.0043 for age as linear trend); KPS 0.0964 (0.2379 for KPS as linear trend); Extra-cranial metastases 0.1719; Primary NSCLC 0.0941; Number of brain metastases 0.3377; RPA 0.2762 (0.0834 for RPA as linear trend); GPA 0.2642 (0.0812 for GPA as linear trend).

**Supplementary Figure S1: Dexamethasone use over time.**

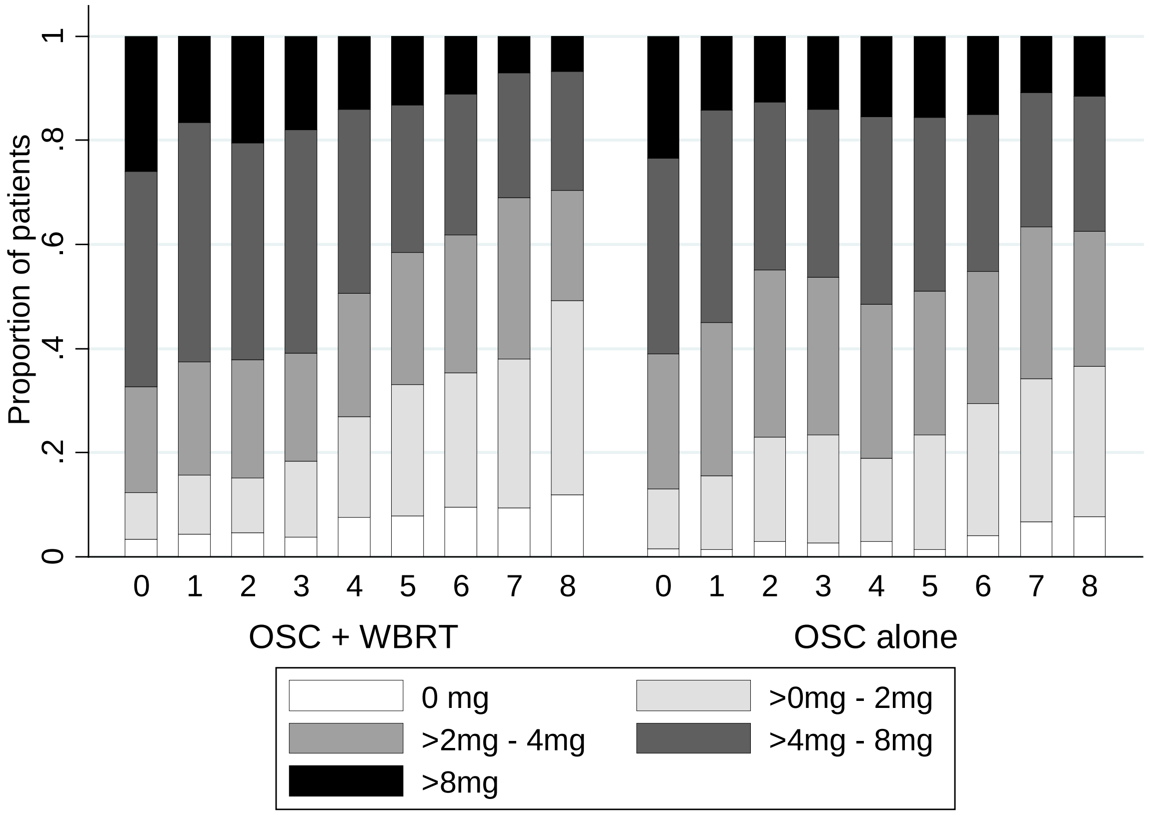


Notes: Week 0 = dose received at baseline.

Number of patients with data at each timepoint (WBRT, OSC):

Week 0 (269, 269); week 1 (211, 218); week 2 (219, 205); week 3 (212, 192); week 4 (186, 175); week 5 (166, 141); week 6 (136, 126); week 7 (129, 120); week 8 (118, 104).

Proportions are calculated from the number of patients with non-missing data.

**Supplementary Figure S2: Time to dexamethasone reduction**

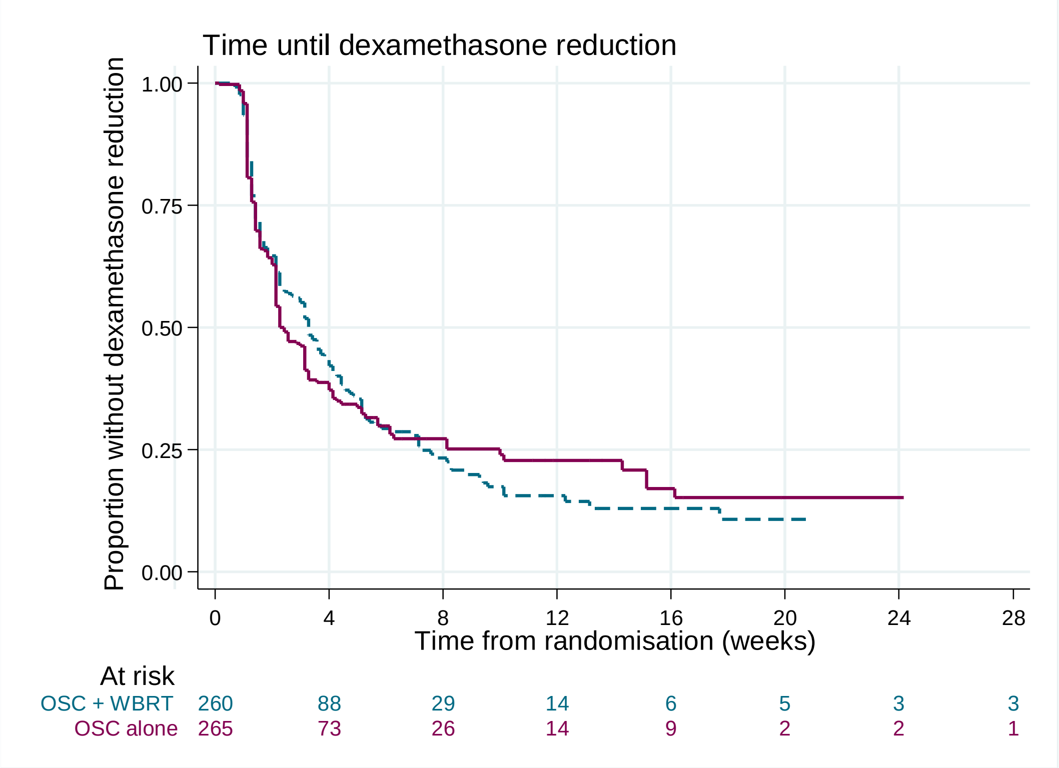

**Supplementary Figure S3: Difference in QALY days, with 90% confidence interval.**

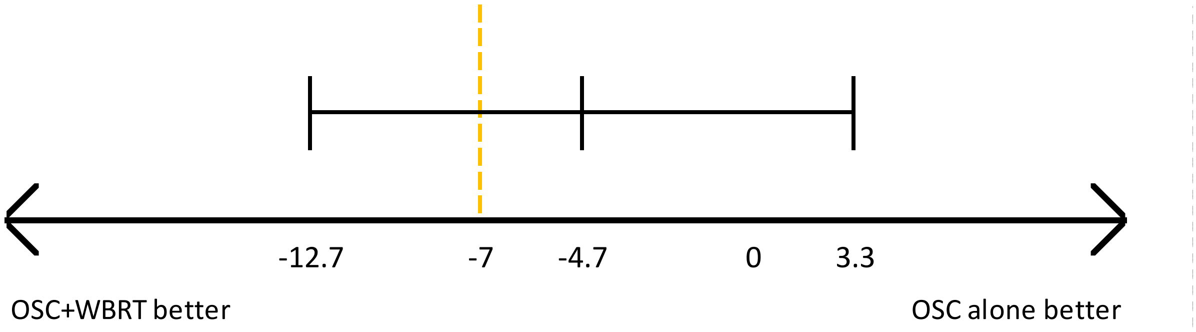


Note: QALY = Quality Adjusted Life Years; OSC = Optimal Supportive Care; WBRT = Whole Brain Radiotherapy.

Note: -4.7 days represents the estimate of the difference in QALYs. -12.7 days and 3.3 days represent the upper and lower limit of the 90% confidence interval for the difference in QALYs (OSC alone – OSC+WBRT).

Note: The yellow dashed vertical line represents the pre-specified non-inferiority boundary of seven days.

ACKNOWLEDGEMENTS

MRC CTU at UCL staff:

Ruth Langley, Lizzie Armstrong, Sarah Beall, Christina Chung, Cheryl Courtney, Sadiq Gearay, Gosala Gopalakrishnan, Lynda Harper, Nadine van Looy, Brendan Mauger, Jacque Millet, Matthew Nankivell, Laura Nichols, Mahesh Parmar, Cheryl Pugh, Annabelle South, Richard Stephens, Ben Sydes, Nancy Tappenden, Barbara Uscinka.

Trial Management Group:

Paula Mulvenna, David Ardron, Rachael Barton, Nicola Bell, Iona Brisbane, Corinne Faivre-Finn, Georgina Gerrard, Helen Jones, Elaine McColl, Barbara Moore, Fiona Richards, Paula Wilson.

Trans Tasman Radiation Oncology Group (TROG):

David Ball, Kacy Baumann, Tanya Holt.

Independent Data Monitoring Committee:

Anna Gregor, Alison Brewster, Bill Noble, Linda Sharples.

Other advisors/collaborators:

Sam Ahmedzai, Jenny Donovan, Lesley Fallowfield, Patricia Fisher, Neal Navani, Carolyn Pitceathly.

Participating Centre Staff:

Aberdeen Royal Infirmary (David Hurman, Donald Bissett, Shelagh Bonner-Shand, Louise Brown, Pat Cooper, Marianne Nicolson, Margaret Ritchie, Sue Rodwell, Ravi Sharma);

Addenbrooke's Hospital (Kathryn Waite, Michele Bianchi, Chloe Bryan, Gill Charman, Anita Chhabra, Mario Coliof, Pippa Corrie, Kate Fife, Alison Geib, David Gilligan, Amy Gladwell, Michael Gonzalez, Vanessa Goss, Debbie Gregory, Yasser Haba, Susan Harden, Roy Harris, Abigail Hollingdale, Ingrid Inman, Vicky Joslin, Lavinia Magee, Claude Magri, Adam McGeoch, Sarah Nolasco, Jo Piper, Yvonne Rimmer, Nicola Robinson, Glynn Rolland, Lois Temel);

Basingstoke and North Hampshire Hospital (Andrew Jackson, Tony Dhillon, Adrienn Fazekasne Fulep, Liz Happle, Catherine Rimington, Jackie Smith);

Beatson West of Scotland Cancer Centre (Graeme Lumsden, Iona Brisbane, Jim Cassidy, Brian Clark, Chloe Cowan, Grainne Dunn, Geri Hamilton, Jonathon Hicks, Calum Innes, Sai Juan Jia, Richard D

Jones, Claire Lawless, Audrey Leonard, Nicky MacLeod, Anne McKillop, Nazia Mohammed, Rebecca Muirhead, Noelle O'Rourke, Kirsteen Stuart, Shilpa Thapar, Aisha Tufail, Cheryl Wilson);

Belfast City Hospital (Jonathan McAleese, Paula McCloskey, Darren Brady, Alison Clarke, Peter Clarke, Prantik Das, Eileen Dillon, Ruth Eakin, Dean Fennell, Emma Gibson, Gerry Hanna, Jackie Harney, Stacey Hetherington, Fionnuala Houghton, Sharon Hynds, Lucy Jellett, Ruth Johnston, Kathleen Mason, Jo McAllister, Bronagh McClory, Karen McKenna, Angela Morrison, Stacey Murray, David Stewart, Stephen Stranex, Yvonne Summers, Jonathan Thompson, Richard Wilson);

Bristol Haematology & Oncology Centre (Paula Wilson, Charles Comins, Sue Cowley, Stephen Falk, Emily Foulstone, Dorothy Griffiths, Elizabeth Harris, Eleanor Hiscott, Robert Hollister, Kirsten Hopkins, Rhiannon Macefield, Carys Morgan, Louise Pope, Helen Saldanha, Mary Simmonds, Beth Thorne, Sue Yarrow);

Castle Hill Hospital (Rachael Barton, Sadie Andrews, Suzy Bunton, Louise Crickmore, Marie D'Arcy, Patricia Dawson, Janet Downs, Paula Gawthorpe, Clair Gillett, Carl Hemingway, Carol Hodson, Jan Hunter, Sadie Millington, Sarah Moffat, Paula O'Reilly, Marian Opoku-Fofie, Pamela Parkinson, Emma Rampling, Lucy Richardson, Karen Shepherd, Karen Stubbs, Clare Swift, Carole Tempest);

Cheltenham General Hospital (Sam Guglani, Susan Anderson, Eleanor Andrews (nee Moore), Rehana Bakawala, Charlie Candish, Lin Crosseley, Sue Davies, Sean Elyan, David Farrugia, Chris Ford, Janet Forkes, Julia Hall, Linda Harris, Nadia Holland, Peter Jenkins, Fiona Jennings, Alison Morgan, Nick Reed, Clive Stokes, Abi Stuart, Catherine Stuart-Grumbar, Kate Trigg-Hogarth, Jayne Tyler, Sue Wronski);

Chesterfield Royal Hospital (Emma Bates, Sheree Hall, Jayaram Mohanamurali, Terri-Ann Sewell, Lesley Stevenson);

The Christie NHS Foundation Trust (Corinne Faivre-Finn, Michael Aylmer, Safia Barber, Neil Bayman, Theresa Breen, Paul Burt, Clara Chan, Ruth Conroy, Sue Dyde, Laura Forker, Tracy Gardener, Rao Gattamaneni, Eileen Hackman (nee Walsh), Margaret Harris, Tony Howell, Pooja Jain, Claire Kelly, Pek Keng-Koh, Caroline Lee, Catherine McBain, Fiona McKirdy, Liz Molloy, Lisa Morris, Tina Pritchard, Mohammed Rashid, Raj Shrimali, Janet Simpson, Nita Smeeton, Alexander Stordy, Priya Sumra, Alison Walker, Gillian Whitfield, Sharon Woolley);

Clatterbridge Centre for Oncology (John Littler, Jennifer Almond, Pam Brereton, Peter Clark, Diane Fildes, Pooja Jain, Laurie Lomax, Zafar Malik, Sarah Rose, Mohamed Saipiliai, Penny Schofield, Asha Sivapalasuntharam, Janet Spriggs);

Clayton Hospital (Julie Ball, Marilyn Bond, Anne Fonseca, Rebecca Foster, Aimee Hayton-Bott, Tracey Lowry, Christine Oxnard, Claire Pratt, Rob Stuart, Katie Wignall);

Cookridge Hospital (Rob Turner, Marilyn Bond, David Bottomley, Kevin Franks, Lynda Garbutt, Georgina Gerrard, James Goulding, Irene Greenwood, Paul Hatfield, Julie Mansell, Fatima Murad, Fiona Richards, Matt Seymour, Mike Snee, Rob Stuart, Johanne Sugden);

Crosshouse Hospital (Pamela Beattie, Philip Cannon, Brian Clark, Jane Clemments, Carrie Featherstone, Lynne Ferguson, Carolynn Lamb, Nicky MacLeod, David McIntosh, Margaret McKernan, Ghazia Shaith, Raj Shrimali, Aisha Tufail, Claudia Turley);

Cumberland Infirmary (Sandeep Singhal, Angela Birt, Joshua Campey-Lancaster, Diane Donnelly, Paul Dyson, Charlotte Eyles, Ivor Hughes, Alva Paterson, Leanne Woodford);

Diana Princess of Wales Hospital (Sunil Upadhyay, Lara Bhaduri, Mohammad Butt, Victoria Ford, Jonathan Hatton, William Holroyd, Maria Newton, Trudy Nurse, Deborah Reed);

Essex County Hospital (Dakshinamoorthy Muthukumar, Devy Basu, Louise Chapman, Hayley Hewer, Philip Murray, Lucy Thorogood);

Forth Valley Royal Hospital (Richard D Jones, Lynn Prentice, Evelyn Smillie, Theresa Thomson, Jennifer Wilson, Sally Young);

Freeman Hospital (Paula Mulvenna, Philip Atherton, Chris Barron, Lucy Blackwell, Michelle Borthwick, Kay Carson, Fiona Devereux, Claire Donaldson, Louise Li, Gavin Mankin, Gourdarz Mazdai, Fiona McDonald, Zarine Razvi, Alison Rowell, Christine Rushton, Vicky Sutton, Jayashree Walker, Nichola Waugh, Bridget Workman);

George Eliot Hospital (Mark Hocking, Sarah Evans, June Jones, Paul Mills, Gordon O'Neil, Rachael Oates, Kay Sanders, Justin Somanathan, Emily Steventon, Jenna Williams);

Glan Clwyd Hospital (Angel Garcia-Alonso, Annette Bolger, Bruce Burnett, Angela Evans, Simon Gollins, Jayne Jones, Joanne Lewis, Carey Macdonald Smith, Jane Stockport, Rachel Thomas, Claire Watkins, Linzi Williams, Fiona Wilson, Cathryn Wood);

Harrogate District Hospital (Joji Joseph, Nicola Bell, Sam Chan, Angela Darby, Kate Gunn, Joyce Guy, Barbara Heath, Helen Lyon, Angela Norton, Nicky Thomas);

Homerton University Hospital (Paula Wells);

Huddersfield Royal Infirmary (Pooja Jain, Barbara Crosse, Linda Bamford, Nicky Daker, Penny Daynes, Lisa Gledhill, James Goulding, Denise Hancock, Lisa Horner, Helen Jones, Adam Mawer, Fatima Murad, Rachel Parker, Kully Sandhu, Rob Turner, Tracy Wood, Emma Woodward, Sharon Woolley);

James Cook University Hospital (Clive Peedell, Adrian Rathmell, Lorraine Atkinson, Eleanor Aynsley, Fathi Azribi, Caroline Brownless, Ruth Carr, Sath Gokul, Mags Harland, Sarah Lawless, Carol Long, Julia McBride, Vanessa McGowan, Jackie Mitchell, Devadasan Shakespeare, Nicola Storey, Emma Thompson, Hans Van der Voet, Andrea Watson);

Kidderminster General Hospital (Mark Churn, Sally Davis, Lorraine Head, Linda Higgins, Pek Keng-Koh, Helen Knott, Rakesh Mehra, Helen Tranter);

King's Mill Hospital (Karen Foweraker, Amy Jordan, Dominic Nash, Susan Smith);

London Road Community Hospital (Kristina Duggleby, Dawn Ennis, Sue Marriott, Dakshinamoorthy Muthukumar, Colin Ward);

Macclesfield District General (Hamid Sheikh, Victoria Adinkra, Karen Clayton, Lorraine Creech, Lisa Hardstaff, Pippa Hill, Mark Lawrence, Nicola Lunt, Marilyn McCurrie, Barbara Townley, Lesley Wilkinson, Iain Woodhouse);

Maidstone Hospital (Timothy Sevitt, Russell Burcombe, Sharon Beesley, Karen Brett, Su Burrage, Clare Calvert, Mathilda Cominos, Helen Coppins, Heather Dias, Nkechi Ebele, Sara Hodgkinson, Victoria Ingleson, Barbara Le Brocq, Ioanna Letsa, Barbara Mercier, Christos Mikropoulos, Innocent Neshiri, Polly Rogerson, Dag Rutter, Claire Ryan, Rachel Ryan, Sarah Stoneham, Henry Taylor, Andrew Visioli, Sandra Wakelin, Pauline Wood);

Mater Hospital (Brisbane) (Tanya Holt, Kacy Baumann, Wendy Hansford, Rebecca Heska, Michael Poulsen, Adrienne See, Sally Whiting);

Mount Vernon Hospital (Jeanette Dickson, Peter Hoskin, Tara Chalk, Joy Dabula, Bernard Delooze, Arshi Denton, Catherine Lemon, Ethan Lyn, Andreas Makris, Peter Ostler, Heather Phillips, Clare Scarlett);

New Cross Hospital, Wolverhampton (Ian Sayers, Ivanna Baker, Nazim Begum, Pauline Boyle, Vanda Carter, David Ferry, Linda Higgins, Baljinder Kaur, Pek Keng-Koh, Christine Kirk, Rakesh Mehra, Kelly Papavarnava, Renita Pawaroo, Kerry Roden, Ali Samanci, Debbie Spruce);

Newcastle General Hospital (Paula Mulvenna, Fiona McDonald, Philip Atherton, Chris Barron, Kay Carson, Claire Donaldson, Louise Li, Gavin Mankin, Gourdarz Mazdai, Rhona McMenemin, Zarine Razvi, Christine Rushton);

Ninewells Hospital (Emma JH Brown, Hannah Lord, Pamela Duthie, Debbie Forbes, Audrey Lyall, Elaine Rankin, Moira Rogers, Alison Smith);

North Tyneside General Hospital (Philip Atherton, Helen Bailey, Alexis Burn, Peter Fenwick, Jill Gardiner, Karen Hurson, Louise Li, Susan Longstaff, Lynn Mann, Tim Peel, Julia Scott, Christina Tanney, Leah Taylor, Steve Williamson);

Nottingham University Hospitals, City Campus (Sally Morgan, Rachel Abbotts, Caroline Atkinson, Julie Berridge, Lauren Blackburn, Alex Blades, Emma Blades (nee Beeton), Louise Brookes, Victoria Brown, Kate Cardale, Michelle Cunnell, Karen Foweraker, Shaymaa Hosni, Kerstie Johnson, Catherine Knox, Raquel Lopez Ramon, Jamie Mills, Abigail Pascoe, Ian Sayers, Carl Sheppard, Catherine Shneerson, Edward Stimpson, Liz Stones, Richard Swinden, Georgina Walker, Ann Worley, Jackie Worville);

Peter MacCallum Cancer Institute (David Ball, Angela Burman, Deborah Cruickshank, Mary Duffy, Danny Duplan, Tsien Fua, Mei Krishnasamy, Michael Macmanus, Nicki Plumridge, Carol Rice, Mark Shaw, Virginia Tuckwell, Greg Wheeler);

Princess Alexandra Hospital (Australia) (Bryan Burmeister, Nancy D'Arcy, Jennifer Suffolk);

Princess Royal Hospital (Hull) (Rachael Barton, Sadie Andrews, Suzy Bunton, Marie D'Arcy, Janet Downs, Paula Gawthorpe, Christopher Hamilton, Jan Hunter, Sarah Moffat, Marian Opoku-Fofie, Pamela Parkinson, Emma Rampling, Lucy Richardson, Karen Shepherd, Kirstie Smith, Clare Swift, Carole Tempest);

Queen Elizabeth Hospital (Gateshead) (Fiona McDonald, Gillian Appleby, Sheila Graham, Amanda Howey, Tracy Lindsay, Susan Longstaff, Zarine Razvi, Julia Scott, Claire Wipat);

Queen Elizabeth Hospital (King's Lynn) (Kathryn Waite, Gwyneth Capes, Verity Connolly, Julie Coyne, Rachel Crown, Margaret Daly, Syed Haidar, Pauline Lingwood, Karen Mayes, Annette Miles, Arleen Parcon, Corrinne Rankin, Sophy Shedwell, Kayleigh Shough, Nicola Ward, Hayley Webb, Jessica Wrigley);

Raigmore Hospital (Carol Macgregor, Florence Anderson, Seonaid Arnott, Sandra Brown, Jane Campbell, Steve Colligan, Kay Kelly, Charles Kodikara, Alison Macdonald, Jude Madeleine, Neil McPhail, Catriona Morrison, Alison Nicholls, Ian Rudd, Azmat Sadozye, Georgina Simpson, Anna Skene, Isobel Stuart, Una Taylor, David Whillis);

Royal Berkshire Hospital (Richard B Brown, Ali Abbas, Joss Adams, Debbie Cartwright, Kristy Coomber, Juliette Dye, Fiona Everson, James Gildersleve, Allison Hunt, Wioletta Kowalczyk-Williams, Christina Lewis, Emma Vowell);

Royal Derby Hospital (Anish Bali, Dawn Ennis, Rajeev Kaushal, Sue Marriott, Dakshinamoorthy Muthukumar, Pugazhenthi Pattu, Colin Ward);

Royal Devon and Exeter Hospital (Elizabeth Toy, John Anderson, Kizzy Baines, Karen Brown, Kiran Das, Elizabeth Davey, Melissa Davey, Tania Davies, Nicole Dorey, Susan Downer, Dawn Edwards, Gail Fielding, Melanie Osborne, Clare Radstone, Alison Roantree, Suzy Tasker, Katie Timmings, Paula Underhill, Elaine Vandcandelaere, Claire Webb);

Royal Marsden Hospital (Sutton) (Imogen Locke, Merina Ahmed, Mike Brada, Karen Brooks, Zahara Ghory, Cordelia Grant, Mark Hill, Alan Horwich, Fiona McDonald, Sally Moore, Lisa Rowland, Elizabeth Sheridan, Priya Yoganathan);

Royal Sussex County Hospital (Geoff Newman, David Bloomfield, Philippa Carr, Karen Cavell, Jane Dexter, Sarah Doffman, Jodie Fleming, Jane Hanson, Samantha Hodges, Simon Hooper, Tamsin Kent, Pauline Martin, Helen Mitchell, Sankha Mitra, Elaine Noon, Lynne Omar, Tenesa Sargent, Richard Simcock, Joanna Simpson, Jean Tremlett, Sue Trotter);

Royal United Hospital (Olivera Frim, Tania Allen, Hannah Blades, Rachael Bolitho, Joanne Botten, Vicki Clarke, Ashley Cox, Christine Cox, Samantha Curtis, Emma De Winton, Olivera Frim, Leonie Harrison, Rachael Howard, Dorothy Kumar, Carey Logan, Katarzyna Machura, Kate Moloney, Sarah Murdoch, Joseph Needham, Vicki Portingale, Annie Taylor, Tom Tylee, Jess White);

Russells Hall Hospital (Pek Keng-Koh, Daniel Bull, Lesley Edwards, Simon Grumett, Kathy Harrow, Karen Kanyi, Lucy Smith, Sara Smith);

Scarborough General Hospital (Rachael Barton, Alison Ames, Donna Anderson, Fizzah Asif, Chloe Box, Joanne Fletcher, Tracey Hawkes, Sacha Honour, Sarah Kent, Polly Needs, Anne Nunn);

South Tyneside District Hospital (Liz Fuller, Joanne Battenbo, Penny Borgen, Alison East, Georgi Georgiev, Chris Jones, Gourdarz Mazdai, Rhona McMenemin, Judith Moore, David Stock, Irene Underwood, Michael Walton);

Southampton General Hospital (Chris Baughan, Adityanarayan Bhatnagar, Andrew Bates, Stefan Brodmann, Lynne Dando, Sajid Durrani, Clare Green, Julie Gwilt, Peter MW Johnson, Vladimir Malykh, Helen Martin, Julie Morris, Christian Ottensmeier, Jocelyn Walters);

Southend University Hospital (Mandip Khaira, Jolly Barber, Olivia Chan, Tim Crook, Juliah Jonasi, Katrina Maitland, Sreekanth Palvai, Jan Prejbisz, Anne CR Robinson, Heather Shires, Colin Trask);

Southern General Hospital (Claire Paterson, Karen Bell, Jackie Byers, Scott Davidson, Janet Devlin, Grainne Dunn, Laura Ferguson, Fiona Maclean, Donna McWilliam);

St Bartholomews Hospital (London) (Paula Wells, Denise Andrews, Marina Baccarini, Nanette Bech-Nielsen, Charlotte Coyte, Dean Fennell, Shahanara Ferdous, Emma Foster, John Gribben, Paul Hillman, Janet Kiff, Antonia Koutsoukou, Samuel Mugari, Alastair Nicholson, Jude Nixon, Catherine Norman, Melanie Scott, Andrew Shaw, Jeremy Steele, Peter Szlosarek, Jonathan Teague);

St James University Hospital (Leeds) (Rob Turner, Gemma Austin (nee Glover), Marilyn Bond, David Bottomley, Rutger Clarke, Kevin Franks, Lynda Garbutt, Georgina Gerrard, James Goulding, Irene Greenwood, Fiona Halstead, Jillian Hanson, Paul Hatfield, Helen Jones, Julie Mansell, Clive Mulatero, Fatima Murad, Fiona Richards, Angela Scotland, Matt Seymour, Mike Snee, Rob Stuart, Johanne Sugden, Kay Tobin);

Stobhill Hospital (Richard D Jones, Karen Bell, Geri Hamilton, Sai Juan Jia, Audrey Leonard, Robert Milroy, Kirsteen Stuart, Claudia Turley);

Tameside General Hospital (Margaret Harris, Michelle Blodwell, Shirley Cocks, Martina Coulding, Edith Curran, Stephanie Ridgway);

Torbay District General Hospital (Nicole Dorey, Nigel Bailey, Victoria Bell, Peter Bliss, Martyn Blundell, Jo Blurton, Geoffrey Cogill, Jenny Cook, Donna Cuffe, Claire Fairfax, Andrew Goodman, Helen Greedus, Ingrid Koehler, Fiona Roberts, Lorraine Thornton, Elizabeth Toy, Linda Welsh, Nick Young);

University Hospital Aintree (John Littler, Lucy Berresford, Chinnamani Eswar, Julie Griffiths, Maria Guerin, Paul Hill, Linda Kearns, Lorraine Lancaster, Haley McCulloch, Leigh Pauls, Shirley Pringle, Sarah Rose, Asha Sivapalasuntharam);

University Hospital Coventry and Warwickshire (Mark Hocking, Maggie Brown, Sue Elwell, Sarah Evans, Kate Field, Alison Franks, Julia Gaston, Sandeep Gill, Bob Grieve, Donna Halliman, Joanna Hamilton, Lesley Hayward, Caroline Humber, Judith Lake, Rosaleen Laverick, Gemma Mansell, Hayley Morton, Efosa Omigie, Pritpal Panesar, Elaine Simmons, Melanie Sinfield, Justin Somanathan, Emily Steventon, Frances White, Kimberley White, Linda Wimbush);

University Hospital Llandough (Jason Lester, Sarah Bridges, Mim Evans, Alison Kelly, Fergus Macbeth, Barbara Moore, Zoe Whale);

University Hospital of North Durham (Rhona McMenemin, Jean Dent, Julie Elliot, Julie Elliott, Jayne McClelland, Lorna Morgan, Dorothy Turnbull, Lisa Wayman, Sarah Welsh);

Velindre Hospital (Jason Lester, Caroline Bobart, Sarah Bridges, Michael Button, Rebecca Cloudsdale, Alison Edwards, Mim Evans, Claire Heymann, Alison Kelly, Fergus Macbeth, Tim Maughan, Barbara Moore, Lynda Penketh, Sara Shankland, Stephen Slade, Helen Stanton, Caroline Vitolo);

Victoria Infirmary (Jonathon Hicks, Karen Bell, Jackie Byers, Janet Devlin, Laura Ferguson, Donna McWilliam, Emma Moody, Rebecca Muirhead, Claire Paterson, Joseph Sarvesvaran, Nicola Steele, Claudia Turley);

Wansbeck General Hospital (Paula Mulvenna, Andrew Johnston, Tessa Lowes, Jeanette Raine, Zarine Razvi, Julia Scott, Marie Todd, Steve Williamson);

Warwick Hospital (Alison Franks, Caroline Humber, Mark Hocking, Michelle Faupel, Lesley Gotschy, Joanna Hamilton, Lyn Hartwell, Julia Jones, Helen Millage, Emily Noonan, Elaine Simmons, Frances White);

West Suffolk Hospital (Yvonne Rimmer, Cherri Blades, Gill Brett, Deborah Clements-Dimmock, Mark Heath, Daniel Patterson, John Raja Ravendar, Christine Watkins);

Weston General Hospital (Waheeda Owadally, Marjorie Tomlinson, Hannah Berry, Christine Graham, Axel Walther, Debbie Coles, Donna Cotterill, Harvey Dymond, Serena Hilman, Sarah Kidd, Denise Leighton-Price, Hugh Lloyd-Jones, Vivienne Pixton, Dawn Simmons, Tom Wells);

Weston Park Hospital (Patricia Fisher, Mymoona Alzouebi, Emma Bates, Laura Borrill, Lesley Bruce, Jaseela Chiramel, Robert Coleman, Sarah Danson, Yvonne Deeley, Julia Disney, Linda Evans, Bernie Foran, Clare Garner, Kate Gibbins, Matthew Hatton, Elizabeth Hodgkinson, Patrick Joyce, Peter Kirkbride, Caroline Lee, Jayaram Mohanamurali, James Swinscoe, Mark Trigg, Penella Woll, Helen Wood, Robin Young);

Whipps Cross University Hospital (Paula Wells, Denise Andrews, Charlotte Coyte, Elizabeth Croydon, Patricia Danaswamy, Emma Foster, Andrew Gillian, Memory Kazingizi, Fiona McKirdy, Samuel Mugari, Denisa Murati, Alastair Nicholson, Thompson Olaoni, Jonathan Teague);

William Harvey Hospital (Russell Burcombe, Thomas Bird, Tracy Boakes, Jane Brown, Julie Buckley, Mathilda Cominos, Denise Crawford, Stuart Drakeley, Susan Drakeley, Louise Gladwell, Coral Greenstreet, Joan Idris, Kathryn Lees, Margaret Lipsham, Sharon Middleton, Rakesh Raman, Karen Robinson, Joanne Severn, Paula Whichelo, Marian Wood, Linda Wray);

Worcestershire Royal Hospital (Mark Churn, Lisa Capaldi, Susan Anderson, Eleanor Andrews (nee Moore), Charlie Candish, Kristy Cleary, Sue Davies, David Farrugia, Janet Forkes, Linda Harris, Jayne Tyler, Sue Wronski);

Worthing Hospital (Nick Adams, Jo Congleton, Angela Dunne, Celia Gonzales, Sarah House, Lynda Huggins, Jayne Hughes, Helen Jones, Geoff Newman, Faye Oliver, Susan Rockall, Kathrine Steele, Nikki Turner, Paula Wakelen);
